# Navigating the interplay between feelings of failure and newfound kindness: a qualitative study exploring the experiences of cancer bereaved fathers

**DOI:** 10.1186/s40359-026-05155-4

**Published:** 2026-07-13

**Authors:** Anna Katharina Vokinger, Eddy Carolina Pedraza, Peter Francis Raguindin, Eva Maria Tinner, André Oscar von Bueren, Katrin Scheinemann, Eva Bergstraesser, Gisela Michel, Eva De Clercq

**Affiliations:** 1 Faculty of Health Sciences and Medicine, University of Lucerne, Alpenquai 4, Lucerne, 6005 Switzerland; 2 Inselspital, University Children’s Hospital Bern, Bern, Switzerland; 3 Pediatric Palliative Care, University Children’s Hospital Zurich, Zurich, Switzerland; 4 Department of Pediatrics, Obstetrics and Gynecology Division of Pediatric Hematology and Oncology University Hospital of Geneva, Geneva, Switzerland; 5 Division of Hematology-Oncology, Children’s Hospital of Eastern Switzerland, St Gallen, Switzerland

**Keywords:** Fathers, Bereavement, Death, Child, Palliative care

## Abstract

**Background:**

The death of a child has a tremendous impact on fathers’ lives. However, their grief experiences remain understudied in pediatric palliative care and childcare research. The aim of this study is to provide insight into bereaved fathers’ experiences following the death of their child to cancer, and to explore their support needs and wishes.

**Methods:**

Semi-structured qualitative interviews were conducted with bereaved fathers. Data collection occurred from March 2022 to September 2023, and reflexive thematic analysis was used for data analysis.

**Results:**

Many bereaved fathers reported a reorientation of self, a shift in their priorities, and newfound kindness towards themselves and others. At the same time, the death of their child also led to feelings of helplessness and failure in their role as fathers. Some fathers expressed a death wish to reunite with their child and some fathers described how they lost the meaning of their lives. Fathers’ grief experiences and coping strategies often differed from that of their partner. In terms of support, many fathers expressed that it was better to have many support options offered to choose from than to have to reach out on their own.

**Conclusions:**

In their bereavement, participating fathers reported shifts in their sense of self and priorities while navigating emotions of failure and newfound kindness. These experiences were compounded by limited access to preferred support. To be adequately cared for, fathers should be given the opportunity to express themselves freely.

**Supplementary Information:**

The online version contains supplementary material available at https://doi.org/10.1186/s40359-026-05155-4.

## Background

The death of a child has a tremendous impact on parents’ lives, causing more severe reactions of grief, including depression, suicidal ideation, anger, and guilt, than the death of other loved ones, such as spouses or parents [1–8]. However, there is a lack of research on the experiences of fathers grieving the death of their child [9–11]. A contributing factor to this lack is that most participants in pediatric palliative care research are mothers [11, 12]. 

Existing research seems to suggest that communication experiences about diagnosis and prognosis [13], as well as grief experiences, support needs, and coping strategies of bereaved fathers and mothers differ [14, 15], with fathers tending to cope in more solitary, stoic and instrumental ways, and mothers adopting more emotional expression strategies, and being able to look for social support [16–18]. These differences may be attributed to fathers’ and mothers’ upbringing and early socialization which can influence their roles in the workforce, childcare and their emotional expressions [9, 19]. Further contributing factors can be gendered assumptions of health and support seeking, such as the understanding that fathers do not talk about their hardships, as they might feel that they are required to be strong and protect their family [20]. 

Recent literature reviews on bereaved fathers indicate that previous studies have largely focused on life-limiting conditions [9, 10]. The loss of a child to cancer may differ from other bereavement experiences due to the uncertainty surrounding the prognosis and the characteristics of the medical interventions. These characteristics include the intensity of treatment and the decision to stop curative treatment. Adding to this is the continued hope that the child can be cured [21, 22], as childhood cancer today has a survival rate of over 85% in Switzerland [23].

This study provides insights into the experiences of bereaved fathers who have lost their child to cancer. Focusing on their grief experiences, coping strategies and perceived support needs and wishes, this study adds to the limited literature on this topic. The findings could aid in developing interventions suited to support cancer bereaved fathers.

## Method

This study is part of a larger project entitled: “Needs, desires and psychosocial outcomes in bereaved parents who lost their child to cancer: palliative and end-of-life care in paediatric oncology” at the University of Lucerne, Switzerland.

### Inclusion criteria and recruitment

The original recruitment included both fathers and mothers [24]. Eligible participants were bereaved parents whose child had been treated for cancer in a pediatric oncology unit in Switzerland. The child had to be diagnosed with cancer at age < 18 years, and > 1 year had passed between the child’s death and the parent’s participation in the study. Eligible parents were informed about the study by the former treating pediatric oncology clinic through the responsible contact person (pediatric oncologist or pediatric palliative care specialist) through an information letter and flyer, or by a Swiss cancer or bereavement support organization via their website or a flyer.

Interested parents could then contact the study team for more information and schedule a date, time and place for an interview. Whenever possible, interviews were not scheduled close to significant dates or festivities such as national or religious holidays and birthdays. Parents interested in participation could decide whether they preferred a joint (with the other parent) or separate interview. Parents were provided with this option to ensure their comfort, while talking about the sensitive topics of their child’s treatment, death and their bereavement experiences.

The presence of a partner frequently serves as a mechanism for shared memory recall, where specific details, timelines, or family events are jointly validated or clarified. Consequently, we acknowledge that the mother’s presence in some of the interviews could have prompted certain reflections and shaped how fathers reported their experiences. This could mean that some accounts in our results represent co-constructed narratives rather than entirely individual paternal reflections.

Due to the sensitive nature of the interviews, we also acknowledge the possibility of participation bias. It is possible that only parents who felt comfortable sharing their experiences participated in this study.

### Data collection and analysis

Data were collected through semi-structured, in-depth qualitative interviews between March 2022 and September 2023. The interviews were conducted in English, French, German, or Swiss German. The interview guide (Appendix 1) was developed by the research team based on a literature review of bereaved parents’ coping strategies [18] and pilot tested. The interviews were conducted on-site at a preferred location of the parents or online. In the case of on-site interviews, all parents wanted to do the interview at home. Before each interview, parents gave their written informed consent to participate and to be recorded. During the interviews, parents could skip questions if they did not wish to answer. Furthermore, parents were advised to take their time if they became emotional. If parents expressed a desire for support after an interview, they were offered to consult a psychologist, who was not involved in the research project. However, none of the parents opted for this.

The interviews were conducted by two female researchers, a senior researcher with a background in philosophy and bioethics (EDC), with extensive experience in palliative care and gender (medicine) research or a PhD student in health sciences with a background in social anthropology (AKV), who is trained in voluntary palliative care companionship. Both researchers had no previous relationship with the study participants.

Before each interview, the researchers explained the purpose of the study. In total, 18 interviews with 23 bereaved parents were completed. For this publication, we analyzed data from the seven interviews that were conducted with bereaved fathers; five were conducted together with the deceased child’s mother and two with the fathers only. Out of the seven interviews, four were conducted at their home and three online via zoom. All interviews occurred once and were audio-recorded. All interviews were anonymously transcribed verbatim using f4transkript transcription software. The Swiss German interviews were transcribed in German. To ensure that the content remained as told by the participants, terms and expressions that only exist in Swiss German, and could not be fully translated into German, were noted in the transcripts. All transcribed records were then transferred to the Atlas.ti 23 and Atlas.ti 24 software for analysis. Braun and Clarke’s six-step approach of reflexive thematic analysis [25, 26] was used by EDC, AKV, GM, a psychologist with expertise in psycho-social oncology and ECP, a health scientist with a background in pediatrics. Data analysis was conducted in the original language of each transcript. After (1) familiarization with the data set and discussion of initial codes (AKV and EDC), (2) inductive coding was performed by going through the transcripts (AKV and EDC). These initial codes were then (3) clustered into potential themes (AKV and EDC). (4) The themes were then retrieved and reviewed, while paying attention to the relation between codes and themes (AKV and EDC). The themes were generated to reflect shared patterns among the datasets. Through this analysis, (5) themes were further defined and refined (AKV and EDC). Steps 2–5 were accomplished by using Atlas.ti 23 and Atlas.ti 24 and by creating handwritten mind maps. These mind maps, which covered the content of the data, the researchers’ ideas and reflections, and possible themes, formed the basis of the research team’s discussions. During discussions, the researchers also recognized and explored their individual interpretations of the data and reflected how their interdisciplinary approach influenced coding and theme development. This process was guided by the reflexivity as described by Braun and Clarke [26], and the hermeneutic understanding that themes are generated through engagement with and interpretation of the data [27, 28]. We hereby also acknowledge that our pre-understandings may have played an integral role throughout the analysis. In the final step, (6) the themes were then incorporated into a report with exemplary quotes, using pseudonymized names for the children. The quotes were translated from French, German or Swiss German into English. To ensure semantic integrity, the research team discussed and verified the translations (AKV, EDC, ECP, GM).

Data saturation was not sought for as it is generally not considered consistent with reflexive thematic analysis [29]. Furthermore, sample adequacy is often determined by “information power” in qualitative inquiry [30]. The aim of our approach within the highly specific target group of bereaved fathers was to obtain rich personal accounts, and meaning was generated through interpretation of the data, which was achieved with the seven in-depth interviews.

## Results

The seven participating fathers had a mean age of 45.3 years (range: 39–55 years) and a mean of 3.1 years (range: 1–8 years) passed since the death of their child. The mean age of the children at diagnosis was 6.1 (range: 3–13) and the mean age at death was 8 years (range: 4–15 years). Three interviews were conducted in Swiss German, two in English, one in French and one in German. On average, the interviews lasted about two hours. Analysis of the interviews revealed four main themes: (1) reorientation of self and priorities: navigating conflicting emotions and newfound kindness, (2) the wish to reunite somewhere, sometime, (3) experienced differences in parental grief, and (4) desire for proactive support.

### Reorientation of self and priorities: navigating conflicting emotions and newfound kindness

The death of their child resulted in a reorientation process of their sense of self and of their priorities. Most fathers reported having to face conflicting emotions of grief and newfound kindness. Reorientation could take on different forms: for many fathers it consisted in a change of priorities, which encompassed a reflection on the amount of time spent at work (vs. the time spent with their families), but also on the kind of work they were involved in (economic gain vs. helping others). For some fathers, these changes to their work lives were rather short-term. Few fathers took a break after the death of their child to travel with their families and find their way back to life: “We flew to a foreign country six months later. […] We knew we needed to recharge our batteries, both physically and mentally” (Father of Bianca). Other work life related changes had long-term impact. For example, a father started working as a caregiver to keep a promise he made to his deceased child and a desire to support others who “actually need” help. Another father, who was already working in the medical field prior to his child’s cancer diagnosis, described a similar feeling of personal fulfillment. He described how he came to understand that his work could contribute to helping other sick children in need. Some fathers who returned to their previous workplace also considered work to provide them structure and diversion – allowing them to temporarily forget their pain. In a parental dyad where the mother was the primary caregiver and homemaker without employment the father expressed how he wished for the mother to also be able to have such a diversion of her pain: “Well, I would have wished it for her [mother] too. Sometimes I went to work and just thought: “I can work now and forget [the pain] a bit and she’s [mother] all alone with this pain"” (Father of Melina). To provide such an opportunity to ease the mother’s pain as well, the father, along with others, supported the mother in finding employment.

Besides changes in priorities, most fathers also expressed doing things for themselves in their free time in solitude, such as visiting the cemetery, going for a run, or doing other physical activities. For some fathers, these activities made them feel close to their deceased child: “[…] the first few months I almost felt like he was running next to me” (Father of Noah).

Many fathers described how they felt they had become more grounded, at ease and kinder to others. For some, this was accompanied by the realization that, prior their child’s death, they themselves did not always know how to respond to difficult events in their social environment. This realization helped some fathers not to react harshly when they were met with improper comments or lack of support in their bereavement. Their own bereavement experiences also led some fathers to become active in peer support groups, and few became event organizers in support groups.

Although fathers were able to experience and express kindness, most reported feeling helpless upon learning of their child’s terminal illness and subsequent death, as there was nothing they could do to prevent their child from dying. For some, this sense of helplessness turned into a sense of failure – that they could not fulfill what they interpreted as “fatherhood”. When their child died, some fathers experienced different feelings. They described a sense of relief that their child was no longer suffering, and that they had lost their feeling of helplessness: “So even today, the fact that my child died is (…) / the bad thing is that he got sick. The fact that he died is good” (Father of Max). A difficulty many fathers encountered after the death was facing their new reality: no longer being able to spend time with their child and having to make their first experiences without their child (Table 1).

**Table 1:** Reorientation of self and priorities: navigating conflicting emotions and newfound kindness (pseudonymized names)

Theme	Quotes
Reorientation and newfound kindness	Father of Martin “I don’t know how to say it because it is so much of cliché but just be kind […] with other[s]. If I can do this [be kind to others] once a day, my day is fulfilled and that’s why maybe I do much, much more than before, many different acts of kindness or just try to be kind. Of course I have [soft laugh] / you still have all the / your habits from the past or you have some things that you had in the past that you want to keep. For a very long time I am fan of music and so I’ll always liked to have this kind of energy from others which can be aggressive which can be everything but I know that we are here together to share something and not to fight between each other [laughing] and it can be from a very calm and soft atmosphere it can be from a lively and aggressive atmosphere where actually people are together because they like the same thing. My focus is much more on these kinds of things.”
	Father of Martin: “[…] My mom has always been scared that I would buy a motorcycle and I respected this and for a very long time, I had only a simple little scooter to go to work but at some point [after Martin’s death] I explained to my mom that I needed an activity for myself to have time myself.”
	Father of Louis: “I work […] to feel useful. To bring money home. To feel good. For lots of things. […] I’m very happy. I’ve been working for six months now. (…) it’s not so that I do not have to go home or to forget or something. It’s to make you feel good. To do things you like. Before, maybe I did something I liked, but not too much. That has changed […] I want to do good for myself. Not harm others. […] I want to follow my path in the best possible way. With kindness and that’s it. No more, and no less. In the sense that I do not want to be a saint.”
	Father of Paul: “To listen to yourself, what is good for you. To not feel obligated because others assume that you have to behave a certain way and [say] “That’s the right way, this is how grief is”. Everyone is really individual [strongly emphasized]. And you should also accept that from your partner, grandparents and siblings. […] That everyone deals with it [death of child] differently and that you can accept this. And that you can also say: “No, I don’t what that”. And in a way, it is difficult, but […] to maintain a good social circle. That’s just so important.”
	Father of Bianca: “I mean, in the end you have to say, the whole topic with faith, in the worst case we die and then there’s nothing after that. But then we have led a life here on earth that has given us meaning, where we have had hope, where we have invested in others and supported people, where we try to add value for others and have not let our heads hang down. That is the worst case and in the best case, we have led exactly this life, and it is true what we believe [heaven]. And it is true. That means we can only win [and reunite with Bianca one day].”
Feelings of failure and relief	Father of Melina: “And as a father, you sometimes feel like a failure. Because you couldn’t protect them from that [death]. But I have to / I have realized that I have to deal with this myself.”
	Father of Max: “Death has only been a relief for us.”
	Father of Noah: “And then he passed away. Why do I say this? I think as soon as you / we felt / you found out there is nothing that can be done. I mean you know there’s going to be an end at some point the longer this drags on, the more difficult it becomes. So yeah, we always felt, I don’t / I hate using lucky or whatever, we were just maybe fortunate that it [child’s illness trajectory] didn’t drag on.”
	Father of Bianca: “When the dying process was initiated, we somehow experienced it so differently. She [child’s mother] was immediately very sad, very exhausted and, as I said, I was somehow on cloud nine. I was simply liberated, because I couldn’t help anyway, now the burden [was taken from me] / because that was one of the worst things, at least for me as a man, because I would have liked to have things under control and if there had somehow been a way to help and that was just not possible and to see that, just that life here was over and I couldn’t do anything and then it was just over (…) / that moment I was no longer helpless.”
Sharing Feelings	Father of Martin: “[…] to share with people. You need to and I think that really depends also, you know, on whether you are surrounded by friends that you trust and find ways to share. If you keep things for yourself, you don’t do anything […] you know, you have all these movies or where the guy is living by themselves and doesn’t share with others but just with nature around them and this brings me back to the sentence of my mom: “Just believe in humans” and that’s where the key lies. If you don’t share, it will just, it’s like a cooking pot - you know - pressure will become so, so, so strong that at some point yeah it doesn’t help. So share, share with people and don’t be scared that sometimes you make mistakes, sometimes you share with someone from whom you don’t receive the expected answer that’s not a problem. You know, you never know if you thought a friend was very strong and would be able to give you support, but he is not. It’s not a problem you just talk about something else then.”

### The wish to reunite somewhere, sometime

Most fathers reported how the death of their child had changed their outlook on life and death. Some fathers reported that at some past point in their grief journey they preferred to stop living because they felt their lives were no longer meaningful.

Some fathers described how they struggled with the thought of how many more years they could have had together and what their child’s future could have been like. A future they would no longer be able to experience together as life ended in the wrong order. In this context, some fathers expressed how it seems as if nothing worse could happen. Some fathers mentioned this in relation to other possible losses, such as the age-related death of their parents. Whilst it would hurt them very much, it scared them less as it would be “part of the path”.

Others expressed a desire to reunite with their children in the future, and some explained how they were no longer afraid of dying but also recognized the value of living here and now. Until they reunite, many fathers described how they want to do their best to be kind to others and to care for the siblings of their deceased child. Some fathers described how they wanted to safeguard the long-term mental health of their other child(ren) by making sure they did not feel “survivor’s guilt”, by giving them the space to grieve in their own way, ensuring they understood what had happened. Caregiving also consisted in few fathers performing everyday tasks, such as making breakfast the morning after their child’s death. Such tasks were described as helping them to keep going and envision the future of their families (Table 2).

**Table 2:** The wish to reunite somewhere, sometime (pseudonymized names)

Theme	Quotes
Wish to reunite	Father of Melina: “I always said / we [mother and father] always said to ourselves: “We actually have two options, either we leave now too, or we carry on as she would have liked to”. That’s what we thought.”
	Father of Bianca: “Later on, this grieving process somehow came up for me / or rather, grief and anger came up for me at a different time. What was important is that we were always open and honest with each other. (…) and that (…) the time period shouldn’t be too long (…) because a lot happens in a short space of time and if things aren’t discussed, then a lot of negative things happen. For example, […] I get to a point where I get angry and don’t want to accept it and don’t communicate it somehow, then something very negative has happened in me, which then has an impact on our relationship, on our whole life. Yes. I suddenly didn’t find this meaningful life so meaningful after all. I wanted everything to be over as quickly as possible and somehow, I wouldn’t have been sad if I hadn’t been alive here anymore. […] I would have preferred not to be here anymore. It’s important that these periods of time are not too long, that we talk to each other, that the other person simply knows where is my partner in the grieving process. That has always been very important in the past and it still is today. That we simply realize that if we do not speak with each other for too long, there is a certain restlessness. […] So our relationship still depends very much on how honest we are with each other and how much time we spend together.”
	Father of Martin: “I mean we have a second child, so we feel about giving them a future. As every parent, we are concerned about the future of the planet and what we will leave to our child and so on. And we would like to, if we can, do something for them and then sure our future and so on. So these are the more / the conventional things [deep breath] and on the other hand maybe, but I think I didn’t reach this point yet […] once I will feel so comfortable that I will say: “Okay, the future, I can live with Martin being at that place right now”. Then I think like okay that’s how I will live: I am concerned for the future for our other child and my concern for the future of Martin is that he is at the right place now. One of my favorite song ends by saying “This isn’t the end” I don’t believe in many things except that humans (…) once it will be my turn, I hope, and I will find Martin again and this is part of my future to join my child again one day.”
	Father of Paul: “Also dying, death. Yes, it is part of it [life]. And sometimes I think: “If we left [died], we would be with Paul now, but now we are here [alive]”. Both / both are beautiful, aren’t they? And of course, you still want to be here [alive], but it gives you a good feeling [deep breath], someone would then also be there [in the afterlife], right?”
	Father of Max: “Someone said “The grief is turning into longing [for the deceased child]. And somehow the grief passes a little, but the longing doesn’t stop”. And I liked that too, you [interviewer] didn’t ask “How do you get over it?” or “How do you get closure? “, but in your question, there was already (…) a / a never-ending process [implied].”

### Experienced differences in parental grief

When reflecting on their bereavement journey, many fathers described differences in the way they and their partners experienced and coped with grief. For some, the different reactions began with the dying process of their child. Some fathers explained how they first felt some relief as the child’s suffering had come to an end but also reported that this relief soon transformed into anger and sadness, whereas for the mother, the sadness and pain began immediately.

In the aftermath of their child’s death, many fathers explained how they sought physical activities, while they perceived that mothers coped by engaging in activities like meditating, reading, or journaling. Some fathers also expressed that they were the ones who were visiting the cemetery more often. In addition, only few fathers sought professional psychological or peer support, while most mothers did.

Some fathers reported that these differences in some cases led to relationship problems, for example when parents could not understand each other’s coping styles. To address possible tension due to misunderstanding, some fathers reported seeking professional advice or using books that provided possible explanations as to how mothers’ and fathers’ reactions to their child’s death may differ and that it is appropriate/normal if they do. This reflection on accepting each other’s reactions and coping styles was done in different ways, some parents started having regular “meetings” to talk about their feelings. Some parental dyads became open to trying out each other’s coping styles, while accepting that they do not have to act in the same way, but that each parent could do what was best for them (Table 3).

**Table 3:** Experienced differences in parental grief (pseudonymized names)

Theme	Quotes
Differences in parental coping strategies	Father of Paul: “She [child’s mother] simply does [copes] a lot more on a spiritual level. Where I tend to (…) not do that. But I actually fully accept that (…). Sometimes you just think to yourself: “Yes, does it have to be like that? " But yes. It’s [coping with grief] just so individual. I’m also different from her and she has to accept the way I do it //.”
	Father of Martin: “A book says that one of the main differences between men and women is the ritual of a man is usually very linked to a specific activity while the ritual of a woman, and I don’t want to fall into clichés, but would be much more to stop and think of something not linked to an activity, it’s not active it’s mental. […] Martin always loved motorcycles and he has been on my scooter (…) so I started this activity in summer [before Martin died] and since then I have kept doing it […] I remember when Martin was behind – you know on the motorcycle you need to grab the person in front of you so you don’t fall down and that is really something that probably kept me going.”
	Father of Noah: “Different ways and I think […] we were each doing it in kind of our own way but it worked and we always encouraged the other “You should try this” and trying sometimes to push it a bit too hard, I guess until we realized that we all go through our own process. […] I think […] we realized that we were both in terms of grief going on a kind of different [path].”
	Father of Martin: “We discussed this a lot with the psychiatrist that follows us. I think we have two different mental strategies due to our different backgrounds, I guess. I have this background […] but this is somebody with another background and another role, that of a mother, maybe she cannot think like this, which I understand, and I respect […]. It’s not because you have a different way of coping that one is right and the other one is wrong […] our doctor told us you are taking two different paths, two completely different strategies but the goal is the same.”

### Desire for proactive support

Fathers expressed a variety of support wishes. Few fathers wanted to talk to other bereaved fathers. However, one found himself the only attendee at a group meeting. In his view, the reason why fathers shied away from meeting peers was that men typically would not want to talk about their hardships with others.

Throughout their child’s illness, all fathers were in close contact with healthcare professionals, and some fathers expressed a desire for ongoing contact. However, several fathers did not receive such ongoing support. This caused additional hardship for some, who felt that while their child was sick, they received a great amount of support from healthcare professionals but none in bereavement.

In general, several fathers expressed a preference for support to be provided by professionals without them having to make contact or search for available support. This approach would allow fathers to accept, or decline offered support. Some fathers expressed how they faced new organizational tasks (e.g. organizing the funeral, writing thank-you cards for those who condoled them, finding support groups), which could be overwhelming. In this regard, a father expressed a desire for a booklet that could provide guidance to parents in this situation. Such guidance could include recommendations to avoid overburdening oneself with tasks and to prioritize self-care (Table 4).

**Table 4:** Desire for proactive support (pseudonymized names)

Theme	Quotes
Support wishes	Father of Louis: “[after the child’s death] You are in another world. So it’s afterwards. We don’t know where we are. We don’t know who we are. And that’s when you really need a helping hand. But yeah, that’s it, not “Do you want me to come?” But rather: “I will and have [emphasized] to come to your house.” See? Because it is this “Do you want…?” there are plenty of people who will say: “No”. But there you have it, after all that, there you have it / “I will come to you”. At least one or two visits in six months, that would be nice.”
	Father of Bianca: “If there would be a booklet with suggestions, don’t make too many plans. So you shouldn’t chase after appointments, but you should somehow have the freedom to say no, but still have the courage to make appointments and meet people. […] Somehow, even after two years, you can start crying, that it’s normal and not somehow the idea that there’s a time when you say, that’s good now, enough time has passed. (…) Maybe a kind of booklet where typical things that occurred to most people, that you just can say: this is a common experience.”

## Discussion

The results of this study illustrate how bereaved fathers navigated the interplay between feelings of failure and newfound kindness, the support they received, and the hardships they went through and continued to experience in bereavement.

Participating fathers reported a reorientation of themselves and their priorities, which they often described as a form of newfound kindness directed both toward themselves and others. This newfound kindness can be understood as a combination of post-traumatic growth, self-compassion and meaning making.

In the context of post-traumatic growth, this kindness aligns with an enhanced capacity to relate to others through compassion and vulnerability [31]. This shift was further sustained by self-compassion as fathers adopted an attitude of gentleness towards their own needs and emotions amidst adversity. As a result, kindness served as a vital counterweight to their feelings of failure. This suggests that self-compassion [32] is not merely a passive outcome, but an active, transformative coping strategy that allowed fathers to reconstruct their paternal identity after cancer-related bereavement. This resulted in changes in employment and new activities. Some of these activities and employments were chosen by fathers as they provided a link to their deceased child or helped to fulfill a promise they made to their child. Changing employments, or doing other activities to make sense of a loss by valuing life or prioritizing family can be defined as “benefit finding” [33], “continuing bonds” or “meaning making” [18] and may arise from a father’s wish to build and continue the legacy of their deceased child [9]. Such coping strategies can also be understood in light of the Dual Process Model of Grief, which suggests that the bereaved oscillate focusing on grief and rebuilding their lives [34]. Our findings highlight that these two processes are closely linked, for example, when fathers reprioritized and reorganized their lives to find meaning in their loss. The ability to find meaning in bereavement can help parents to mitigate the severity of their grief symptoms and facilitate adjustment after their loss [33]. This is of importance as previous studies show that bereaved parents are at risk for anxiety, depression, suicidal ideation and suicide [3–8]. 

Suicidal ideation is defined as “any self-reported thoughts of engaging in suicide-related behavior” [35] and death wish or desire for death, as also expressed in our study, can be considered as a passive version of suicidal ideation [36]. In the present study we distinguished between participating fathers who, at a past point during their bereavement, wished to die in order to be with their child, and those who no longer feared death. The latter group included fathers who reported how they looked forward to reuniting with their child one day, while placing the reunion in the distant future. The desire to reunite can be understood as a way of coping through continuing bonds. By believing in a deceased loved one’s existence in an afterlife, a reunion becomes possible upon one’s own death. This understanding could help to consider death as only a temporary separation [37]. 

After their child’s death, some fathers in this study took a break from work, others returned to their previous workplace shortly after their child’s death as it allowed them to temporarily ease some of their pain. This can be seen as prioritizing what feels best to them – a form of kindness towards themselves. Such a quick return to work can also be a coping strategy, defined as “keeping busy” [18]. Although this may suggest that some fathers would be reluctant to share their feelings and would want to isolate themselves, similar to the findings in a recent review [9], our study shows that it was common among participating fathers to express their feelings. These findings stand in contrast to previous portrayals of paternal bereavement, which often emphasized a tendency toward stoicism, characterized by emotional restraint and a focus on rational self-regulation [9]. While previous literature suggests that such stoic expectations can lead to self-isolation or refraining from sharing vulnerability, the fathers in our study described a different trajectory. They emphasized the importance of active emotional engagement by deliberately participating in family life and practicing what they described as newfound kindness toward their friends and families. This suggests that for this cohort, the experience of grief involved a more expressive and relational form of fatherhood.

While some fathers in our study perceived a general reluctance among their peers to discuss hardships and attributed this to so-called traditional coping roles that still impose a pressure on men to refrain from public vulnerability, the apparent absence of fathers in these public spaces may not solely be a result of traditional gender norms. Instead, it may point to a structural misalignment given that the traditional format of bereavement support groups may not currently meet the specific preferences or needs of fathers. As seen in the present results, participating fathers used more active coping strategies, therefore, a meeting where bereaved fathers are active together, such as going for a run, might be more suitable. An example of a physically active support group is the Sands United football group [38], which was started in the United Kingdom to bring bereaved fathers together and provide a space for them to bond and break the silence of loss [38]. 

Bereavement support can also be provided by family, social environment and healthcare professionals [39]. However, participating fathers reported how appropriate support is often limited. Besides limited availability, isolation from previous support, such as no longer being at the hospital, has been identified as a possible risk factor that can increase parental grief [40]. Healthcare professionals were identified by the fathers as playing a highly important role and have been shown to help alleviate bereaved parents’ hardships [22, 41, 42]. Such an intervention could integrate the Illness Beliefs Model [33] providing healthcare professionals a framework to create open conversations. This could help fathers communicate feelings, recognize their needs as well as accepting possible parental differences in bereavement reactions and overall help to ease the father’s suffering [33]. Recognizing possible parental differences in bereavement could enrich the parents' relationship and prevent further distress [15]. When considering possible differences in bereaved parents’ coping strategies, the multiple roles and facets of parenthood should be taken into account, as these may influence parents’ coping strategies. These include parents’ economic and caregiving roles [18]. While navigating and accepting differences could enrich a relationship, there are also similarities to acknowledge. A recent study on mothers of children in hospice at home also showed that mothers presented feelings of guilt for being unable to protect their child from inevitable death and having to face the reversed natural order [43]. 

To provide access to support, it is of high importance that fathers feel included in the available support. To investigate fathers’ experiences further, researchers should address fathers directly with “Dear mother and father” [10]. Whilst this approach could help including some fathers, it could also lead to the exclusion of non-heterosexual parents. Therefore, future research should investigate how bereavement care services can become more inclusive.

### Clinical implications

Our findings emphasize how bereaved fathers may openly share their emotions. However, fathers in our study sometimes lacked the support they needed to do so effectively. Findings further suggest that we must pay close attention to fathers’ expressions about death wishes. These expressions might be an indication of suicidal risk. However, as distinguishing between a death wish and suicidal ideation is difficult, we recommend that support is available to everyone to identify and address potential red flags.

Furthermore, based on our results and in line with a recent study [42], we propose that support should be offered and made available to fathers, rather than fathers having to seek out support for themselves. Proactive support would allow for fathers to choose what suits them best, while they tackle and navigate all the other challenges they face following their child’s death. Such proactive support could include a follow-up contact that is provided to every bereaved parent, which acknowledges possible similarities and differences within parental couples’ coping strategies. During such follow-ups, parents could be provided with written information on where to find suitable additional support.

In regard to the possible type of offered support we propose more activity-based options to be made available, such as the Sands United Football Club [38] or running clubs. Additional support should be initiated by the former healthcare and psychosocial team that was involved in the child’s care. This support should aim to ease fathers’ sense of failure by helping them transform their “failure” or “defeat” into kindness towards themselves and others, which could possibly foster post-traumatic growth.

To further tackle the issue of fathers lacking access to support services and feeling excluded, they should be addressed directly and included in the development of new programs. Furthermore, healthcare professionals and researchers should reflect on their own perceptions of parental roles and not assume stereotypical needs. By doing so, they can ensure that parents have the opportunity to express themselves freely, without being constrained by the roles and behavior attributed to them.

### Strengths and limitations

It proved challenging to recruit fathers and our data collection therefore consists of a limited sample of seven participating fathers. A further limitation is our rather homogenous sample consisting of heterosexual, white men. Two female researchers collected the data, and this might have influenced the interactions and information shared by fathers. Furthermore, we cannot rule out participation bias: the fathers who participated in the interviews might be more communicative and open about talking about the loss of their child than those who did not participate. Additionally, interviews conducted with both parents together might have influenced the content and narrative of the interviews.

Strengths of this research are consistency between the data presented and the findings, and the diverse specializations of the research team. An additional strength is how the participating fathers spoke very openly and extensively about their experiences resulting in very rich data.

## Conclusion

In their bereavement, participating fathers reported shifts of their sense of self and priorities, as a form of newfound kindness towards themselves and others. Throughout their grief journey, fathers sought/needed specific support and expressed a variety of emotions. These included feelings of failure and helplessness as they could not save their child and the desire to be reunited with their child. Compared to previous studies, the participating fathers more openly shared their emotions and seemed less stoic. Therefore, it is important that bereaved fathers are given opportunities to freely express themselves to be seen, heard, and cared for.

## Supplementary Information

Supplementary Material 1.

## Data Availability

The authors can provide additional information about the data upon request. However, data are not publicly available due to ethical and privacy restrictions. Please contact the corresponding author.

## Abbreviations

(…): Break
[…]: Deleted content
[content]: Additional content provided for readers’ understanding
/: Abrupt stop within a sentence
