# Supplementary material for: Navigating the interplay between feelings of failure and newfound kindness: a qualitative study exploring the experiences of cancer bereaved fathers
Source: BMC Psychol. 2026 Jul 13;14:1447. doi: 10.1186/s40359-026-05155-4 (PMC13643957; doi:10.1186/s40359-026-05155-4)
Supplement: Supplementary file 1 [file 40359_2026_5155_MOESM1_ESM.pdf]

Bereavement Study: Interview Guide

<table>
<tr><td>Introduction</td><td>Explanation regarding the project and why we find it important to address this topic.</td></tr>
<tr><td>Narrative of the lived experience</td><td>I am interested in learning more about the life stories of people who lost their child to cancer.

I would like to listen to your story, I want to know more about your experiences in the period prior to your child's passing, at the time of death and in the period after your loss.

I am interested in your needs, your support networks over time, experiences and events that were particularly meaningful for you and your family.

While I have some questions to guide us along in the interview process, I am interested in hearing about anything else that you think would be useful to my understanding.

As we go through the interview, there may be questions that you would rather not answer. In this case, please feel free to "pass" on that question. Also, if any question is unclear, don't be afraid of telling me so. I will do my best to ask it more clearly.

Please be assured that your responses and all the information/data you provide are completely confidential and anonymous. Your participation in this research study is voluntary. You may withdraw at any time and for any reason without an explanation.

While listening I might be taking some notes to ask you some follow-up questions. However, to be fully engaged in the interview and for research purposes, I would like to audio-record our conversation. Is that alright with you?

Then just one last reminder: there are no right or wrong answers; I am only interested in listening to what you have to say.</td></tr>
<tr><td>Interview guide:</td><td></td></tr>
<tr><td>Icebreaker

On the time leading up to death</td><td>Before we begin, I would like to get to know your family a little better, so I know who you are talking about during the interview.

Can you tell me a bit about X (name of child who died from cancer)?

Can you tell me a bit more about how you experienced the time leading up to your child's death?
<u>Follow-up questions:</u>
- How and when did you become aware of the fact that your child's illness was terminal?
Follow-up:
- By whom were you informed?
- Were you satisfied about the way you were informed about the prognosis (content & manner)?
- Was your child (ask: in case of adolescent) present during these discussions?
- Did you inform your child about it? (if yes: Can you tell me a bit more when and how you discussed the topic of death?)</td></tr>
</table>

	- Whom else did you inform about your child's prognosis? When and how did you inform them?<br>- How did the information you received influence your path?<br>- Was there a moment that was especially significant or stands out for you, for some reason or another, and if so, could you tell me about it?<br>- Do you recall any negative and/or positive experiences?
Support<br><br><br><br><br><br><br><br><br><br><br><br><br><br><br><br>Needs	**Can you walk me through the type of support you received during this challenging time?**<br><br>Follow-up questions:<br>- Personal support: e.g. partner, family members, friends<br>- Professional support: e.g. HCPs (hospital or external services)<br>- Community support: e.g. other affected parent, groups/associations, spiritual support<br>- Did you look for online support? (e.g. Facebook groups?)<br>- Others<br><br>**Looking back, is there anything that you would have changed in terms of the support you received?**<br>Follow-up question:<br>- Is there any support you have not found but you wish existed?<br><br><br>**Based on your response, it seems as if the type of support you received during this time – (did not/partially/did) correspond to your needs. Can you describe me a bit in more detail what kind of needs you had in this time period?**<br>Follow-up questions:<br>Type of needs:<br>- Communication needs (content and form/manner)<br>- Information needs (e.g. respite care, palliative care, dying process)<br>- Emotional needs (e.g. compassionate gestures, attentive listening, kindness, solidarity)<br>- Meaning making needs (e.g. reflecting on meaning and purpose, developing spirituality, making sense of loss, engaging in mindfulness)<br>- Practical needs (e.g. financial assistance, domestic help, the availability of accommodation, medical home-help)<br>- Communication needs (e.g. how to talk to the child, siblings, family)<br>- Existential needs<br><br>- From whom did you wish to receive support in alleviating these needs?<br><br>**Looking back, is there anything the healthcare team could have done differently in terms of support?**
Death and grief (then)	**Can you tell me a bit more about how you experienced the time immediately after your child's death?**<br>Follow-up questions:<br>- Was there a moment that was especially significant or stands out for you, for some reason or another, and if so, could you tell me about it?

	- Do you recall any negative and/or positive experiences?<br>- What helped you most during this time?
Support (services)	**Can you walk me through the type of support you received during this challenging time?**<br><u>Follow-up questions:</u><br>- Personal support: e.g. partner, family members, friends<br>- Professional support: e.g. HCPs (hospital or external services)<br>- As support, some parents and other relatives of the deceased child use services offered outside the hospital. Have you sought outside support?<br>- Community support: e.g. other affected parent, groups/associations, spirituality<br>- Did you look for online support? (e.g. Facebook groups?)<br><br>**Looking back, is there anything that you would have changed in terms of the support you received?**<br><u>Follow-up question:</u><br>- Is there any support you have not found but you wish existed?
Needs	**Based on your response, it seems as if the type of support you received during this time – (did not/partially/did) correspond to your needs. Can you describe me a bit in more detail what kind of needs you had in this time period?**<br><u>Follow-up questions:</u><br>Type of needs:<br>- Communication needs (e.g. how to talk with partner, siblings)<br>- Emotional needs (e.g. compassionate gestures, attentive listening, kindness, solidarity)<br>- Meaning making needs (e.g. reflecting on meaning and purpose, developing spirituality, making sense of loss, engaging in mindfulness)<br>- Practical needs (e.g. organization of rituals, commemoration services etc.)<br>- Information needs (e.g. where to find support)<br>- Existential needs<br><br>- From whom did you wish to receive support in alleviating these needs?
Needs & support for family members	Did your other family members (siblings/ grandparents) need and receive professional bereavement support?<br><br>**Looking back, is there anything the healthcare team could have done differently in terms of support?**

Impact	**Loss has a great impact on our lives. I would like to know how the death of your child affected you and your family.**<br><br>**How would you describe the impact of your child's death on your life immediately afterward X (name of child) died?**<br>Follow-up questions:<br>- Private life/personal relationships<br>- Mental health<br>- Physical health<br>- Professional life/work<br><br>**How would you describe its impact on your life now?**<br>Follow-up questions:<br>- Private life/personal relationships<br>- Mental health<br>- Physical health<br>- Professional life/work
Coping with grief (past-present)<br><br><br><br><br><br><br><br><br><br><br><br><br><br>Suggestions for other parents	**In this part I would like to know more about your coping process – how you and your family tried to manage with your loss.**<br><br>**Can you tell me about what helped you coping with grief during the first days after your child died? (e.g. legacy building, rituals, photobook etc.)**<br><br>**Can you tell me about what helps you to cope with grief now? (e.g. legacy building, rituals, photobook etc.)**<br><br>**Has your grieving experience changed over time (i.e. when your child died compared to now)? Can you explain a bit more in what way/or how?**<br><br>**Did you notice any difference between your grieving experience and that of your partner/your child's sibling or other family members?** If so, can you briefly talk about it?<br><br>**What suggestions would you give to parents to help them cope with grief**<br>- Shortly after the child's death<br>- In the long term (long-term) grief
Closing questions	**About today: Could you tell me about your life now? How do you feel now?**<br>Follow up: Could you tell me about your everyday life? – e.g. what was yesterday like?<br><br>**About the future**: Could you tell me about how you feel regarding the future? (Has your child's death changed your view on life/death?)<br><br>**Is there anything that I have not asked about your bereavement experience and the support you have found or would like to find, that you would like to share?**<br><br>I would like to finish this interview by asking you to share one of your most cherished memories of your child.
